# Peripheral eosinophil counts predict efficacy of anti-CD19 CAR-T cell therapy against B-lineage non-Hodgkin lymphoma

**DOI:** 10.7150/thno.54546

**Published:** 2021-03-04

**Authors:** Qingzhu Jia, Diyuan Qin, Feng He, Qichao Xie, Zhitao Ying, Yajing Zhang, Yuqin Song, Jia-Nan Cheng, Xuejiao Zuo, Luxiang Xu, Hongliang Fang, Chunyan Hu, Lina Peng, Tao Jin, Zixiao Shi, Peter B. Alexander, Yongsheng Wang, Yarong Liu, Weidong Han, Jun Zhu, Pin Wang, Qi-Jing Li, Bo Zhu

**Affiliations:** 1 Department of Oncology, Xinqiao Hospital, Army Medical University, Chongqing 400037, China.; 2 Chongqing Key Laboratory of Immunotherapy, Chongqing 400037, China.; 3 Department of Thoracic Oncology, State Key Laboratory of Biotherapy and Cancer Center, West China Hospital, Sichuan University and Collaborative Innovation Center, Chengdu 610041, China.; 4 Department of Immunology, Duke University Medical Center, Durham, NC, USA.; 5 R&D Department, HRAIN Biotechnology Co. Ltd., Shanghai, China.; 6 Department of Oncology, The Third Affiliated Hospital of Chongqing Medical University, Chongqing 401120, China.; 7 Key Laboratory of Carcinogenesis and Translational Research (Ministry of Education/Beijing), Department of Lymphoma, Peking University Cancer Hospital & Institute, Beijing, China.; 8 Department of Bio-therapeutic, The First Medical Center, Chinese PLA General Hospital, Beijing, China.; 9 Department of Biomedical Engineering, University of Southern California, Los Angeles, CA, USA; Department of Pharmaceutical Sciences and Pharmacology, University of Southern California, 3710 McClintock Ave., RTH506, Los Angeles, CA 90089, USA.

**Keywords:** biomarker, B-NHL, CAR-T, eosinophil, infiltration

## Abstract

**Rationale:** The onset of cytokine release syndrome (CRS) and *in vivo* persistence of anti-CD19 chimeric antigen receptor T (CAR-T) cells after infusion correlate with clinical responsiveness. However, there are no known baseline biomarkers that can predict the prognosis of patients with B-lineage non-Hodgkin lymphoma (B-NHL). The aim of this study was to identify blood cell populations associated with beneficial outcomes in B-NHL patients administered CAR-T cell immunotherapies.

**Methods:** We enumerated peripheral blood and CAR-T cells by retrospectively analyzing three CAR-T cell trials involving 65 B-NHL patients. We used a preclinical model to elucidate the eosinophil mechanism in CAR-T cell therapy.

**Results:** During an observation period up to 30 mo, B-NHL patients with higher baseline eosinophil counts had higher objective response rates than those with low eosinophil counts. Higher baseline eosinophil counts were also significantly associated with durable progression-free survival (PFS). The predictive significance of baseline eosinophil counts was validated in two independent cohorts. A preclinical model showed that eosinophil depletion impairs the intratumoral infiltration of transferred CAR-T cells and reduces CAR-T cell antitumor efficacy.

**Conclusion:** The results of this study suggest that peripheral eosinophils could serve as stratification biomarkers and a recruitment machinery to facilitate anti-CD19 CAR-T cell therapy in B-NHL patients.

## Introduction

Anti-CD19 CAR-T therapies produce durable antitumor responses against B-cell acute lymphoblastic leukemia (ALL) with complete response (CR) rates over 90% 1. However, a major challenge for ALL is that approximately one-third of patients who initially respond relapse within six mo 2. Recently, Fraietta et al. found that CD27^+^CD8^+^CD45RO^-^ CAR-T cells exhibit memory-like characteristics after infusion. Moreover, the baseline percentage of this population in infused CAR-T cell products correlates with sustained disease remission 3. This suggests that a biomarker associated with the persistence of infused CAR-T cells may directly predict the magnitude of chronic lymphocytic leukemia (CLL) responses.

For patients with B-NHL, the best overall response rate to anti-CD19 CAR-T cell therapy is 83%, with only 58% of patients showing a CR after infusion 4, 5. Meanwhile, due to the complexity of treatment, this promising therapy is expensive. Therefore, it is critical to establish rational screening criteria that can be used during baseline assessments to identify superior responders to CAR-T cell immunotherapies.

For anti-CD19 CAR-T cell therapy, a major difference between ALL and B-NHL is that the latter, like a solid tumor, has a microenvironment that can suppress T cell infiltration and activation 6. We hypothesized that a rational baseline biomarker should functionally promote intratumoral T cell responses. Recently, the cotransfer of *in vitro* activated eosinophils has been shown to be correlated with improved efficacy in mouse models evaluating adoptive T cell transfer therapy due to the secretion of the well-known T cell attractants CXCL9 and CXCL10 7. Thus, we examined the clinical association between peripheral eosinophils at baseline and anti-CD19 CAR-T cell efficacy in patients with B-NHL.

## Results

### Patient characteristics

**Figure 1:**
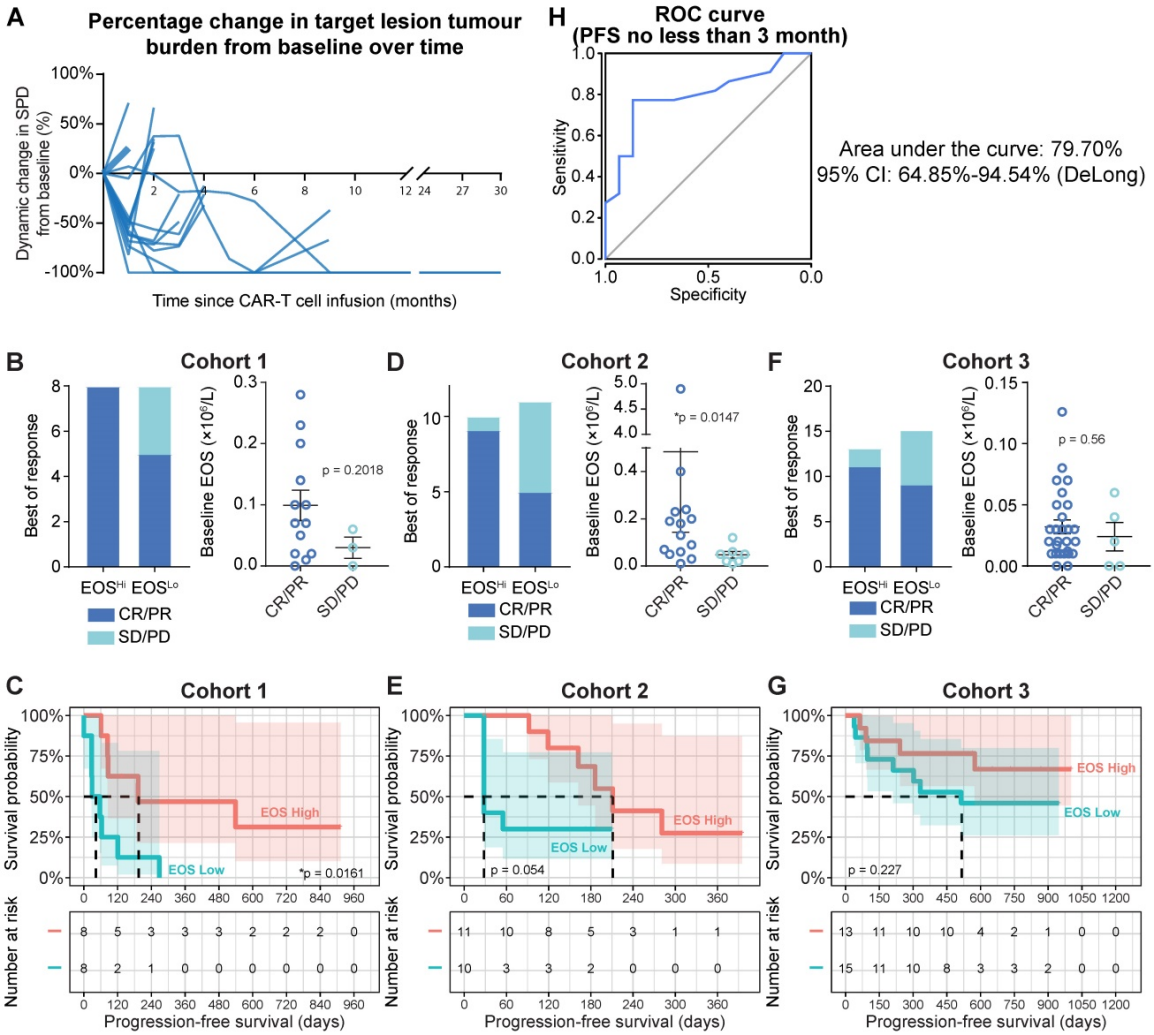
** Therapeutic efficacy of anti-CD19 CAR-T cells. (A)** Tumor burdens were calculated as the sum of the product of the perpendicular diameters (SPD) of all target lesions in Cohort 1 (Discovery Cohort). Horizontal lines indicate no change in tumor burden. **(B)** Left panel: stacked bar graph shows the best objective response (BOR) in patients with high and low baseline eosinophil counts in Cohort 1. Cutoff: 0.07 × 10^6^/L eosinophils, which was the median for this cohort. PD, progressive disease; PR, partial response; CR, complete response. Right panel: Peripheral blood eosinophil counts of patients with favorable and unfavorable BORs in Cohort 1. Statistical analysis was based on the Mann-Whitney *U* test. **(C)** Progression-free survival (PFS) of patient subgroups with high and low eosinophil counts. Tick marks indicate censored patients. The hazard ratio (HR) was calculated by log-rank method. The number at risk is labeled below. **(D, E)** Clinical relevance of baseline eosinophil counts of patients in Cohort 2 (Validation Cohort 1). **(F, G)** Clinical relevance of baseline eosinophil counts of patients in Cohort 3 (Validation Cohort 2).** (H)** Receiver operating characteristic (ROC) curve plotted with the sensitivity and specificity of eosinophil counts for predicting ≥ 3-mo progression-free survival (PFS). The area under the curve (AUC) and confidence interval (CI; calculated by the DeLong method) are shown.

The characteristics of the patients in the Discovery Cohort (Cohort 1) are summarized in **Table S1 and Figure S1A**. In Cohort 1, anti-CD19 CAR-T cells were administered to 16 patients with CD19-positive B-NHL. Thirteen patients were diagnosed with diffuse large B-cell lymphoma (DLBCL), two patients presented with follicular lymphoma (FL), and one patient had marginal zone lymphoma (MZL). All patients except one had progressive disease (PD) at the time of anti-CD19 CAR-T cell infusion. All patients relapsed after ≥ 2 lines of chemotherapy. The median prior chemotherapy regimen comprised nine cycles and three lines of treatments (between 6 to 19 cycles of chemotherapy; 2 to 5 lines of treatment regimens). Five patients had undergone prior radiotherapy. One patient experienced rapidly PD while awaiting T cell infusion. Therefore, short-term palliative radiation therapy (15 Gy/5F, day -7 to day -3 before CAR-T cell infusion) was administered to prevent potential suffocation due to submandibular node progression. Routine follow-up assessments were conducted based on our Institutional Review Board (IRB)-approved protocol (**Figure 1A**).

### Peripheral eosinophil counts correlate with clinical anti-CD19 CAR-T cell efficacy

Tumor progression was assessed by positron emission tomography-computed tomography (PET-CT) or computed tomography (CT) with contrast. Peripheral blood samples were analyzed on days 1, 4, 7, 14, and 28 during the first mo after infusion, every mo for 6 mo, every 3 mo for 2 y, and every 6 mo after the 2-y follow-up. White blood cell counts were obtained by standard clinical laboratory examinations. Focusing on the baseline (blood sample collected immediately prior to preconditioning chemotherapy) blood cell counts, we found that patients with higher (defined as higher than the median value of eosinophil counts, cutoff = 0.07×10^6^/mL) baseline eosinophil levels showed substantially higher objective response rates (**Figure 1B, left panel**; 100% for higher eosinophil counts vs. 62.5% for lower eosinophil counts). We also identified a consistent trend toward favorable clinical responses in patients with high eosinophil levels by comparing baseline eosinophil counts in patients who achieved an objective response (CR and partial response (PR)) or experiencing stable disease (SD) or PD. However, statistical significance was not achieved, as the patient sample size was too small (**Figure 1B, right panel**; 0.099 × 10^6^/mL for CR/PR vs. 0.03 × 10^6^/mL for SD/PD; *P* = 0.2018). Compared to patients with in the subgroup with lower eosinophil counts before infusion, progression-free survival (PFS) was significantly improved (**Figure 1C**; hazard ratio (HR) = 0.3105; 95% confidence interval (CI) = 0.0982-0.9817, *P = 0.0161) in the subgroup with higher eosinophil counts relative to the median. In contrast, the abundance of neutrophils, basophils, lymphocytes, monocytes, platelets, and erythrocytes was not correlated with PFS in this cohort (**Table S2**). Two validation cohorts were introduced as additional supportive evidence. We confirmed the predictive significance of baseline eosinophil counts by applying the median eosinophil count as a cutoff in these validation cohorts (**Figures 1D-1G**; patient characteristics are summarized in **Table S1** and **Figures S1B and 1C**). We consistently found correlations between eosinophil counts and clinical outcomes of CAR-T cell therapies, namely, higher objective response rates (**Figure 1D**: 90.0% for higher eosinophils vs. 45.5% for lower eosinophils; **Figure 1F**: 84.6% for higher eosinophils vs. 60.0% for lower eosinophils) and durable survival benefits (**Figure 1E**: HR = 0.410; 95% CI = 0.128-1.312; *P* = 0.0545; **Figure 1G**: HR = 0.486; 95% CI = 0.1568-1.508; *P* = 0.2273) for patients with high eosinophil levels compared to those with low eosinophil levels. A multivariate Cox proportional model identified the baseline eosinophil count as an independent predictor of CAR-T cell therapy efficacy (**Table S3**).

We plotted a receiver operating characteristic (ROC) curve 8 based on the merged cohort and found that peripheral eosinophil counts effectively predicted durable PFS (**Figure 1H**; ≥ 3-mo PFS; area under the curve (AUC) = 79.70%; 95% CI = 64.85-94.54%) for patients treated with anti-CD19 CAR-T cell therapy.

### Peripheral eosinophil surge precedes tumor pseudoprogression and clinical response to anti-CD19 CAR-T cells

**Figure 2:**
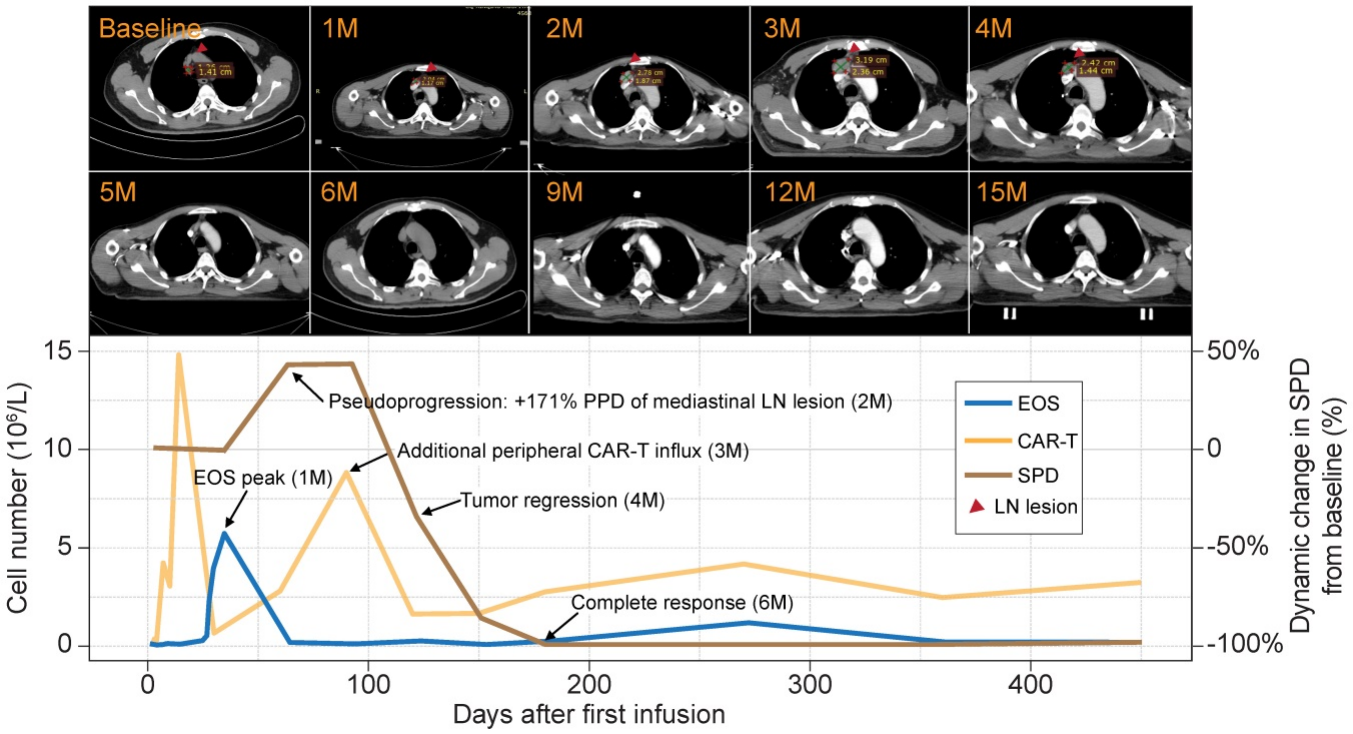
** Peripheral eosinophil influx precedes pseudoprogression and a durable antitumor response in a B-NHL patient.** Upper panel: surveillance CT scans show changes in mediastinal lymph node tumor burden over time. Red arrowheads point to lesion site. Lower panel: clinical course of a patient who experienced tumor pseudoprogression followed by remission. PPD, product of the perpendicular diameter. Peripheral eosinophil counts, CAR-T cells, and SPD for this case are plotted.

Patient 8 was a male aged 58 y presenting with stage IIIA chemotherapy-refractory FL. He received an infusion of 4.5 × 10^7^ anti-CD19 CAR-T cells (7.5 × 10^5^ CAR-T cells/kg) that were intravenously administered as a single dose. Despite showing SD at the first follow-up, the patient exhibited PD in the two following surveillance scans. The sum of the product of the perpendicular diameters (SPD) 9 indicated that the tumor was 1.5× larger than at baseline. The lesion rapidly shrank, and the patient eventually achieved complete remission at 6 mo postinfusion. The remission was sustained for > 30 mo, and there were no apparent radiological or clinical disease symptoms (**Figure 2, upper panel**).

Flow cytometry analysis of the peripheral blood of this patient revealed two significant CAR-T cell influx peaks. The first appeared at 14 days postinfusion and was typical of most patients. However, an uncommon second peak appeared at 3 mo postinfusion, immediately before the remission of the tumor burden. A review of the blood cell counts identified dramatic eosinophilia before tumor pseudoprogression and CAR-T cell influx. At that time, the eosinophils were 9× higher than at baseline and eventually constituted 53.7% of the total white blood cell count (**Figure 2, lower panel**). The sequential timing of these clinical events suggests putative functional relationships among eosinophilia, CAR-T cell activation, and tumor lesion eradication.

### Eosinophil depletion impedes CAR-T cell antitumor efficacy in a preclinical lymphoma model

**Figure 3:**
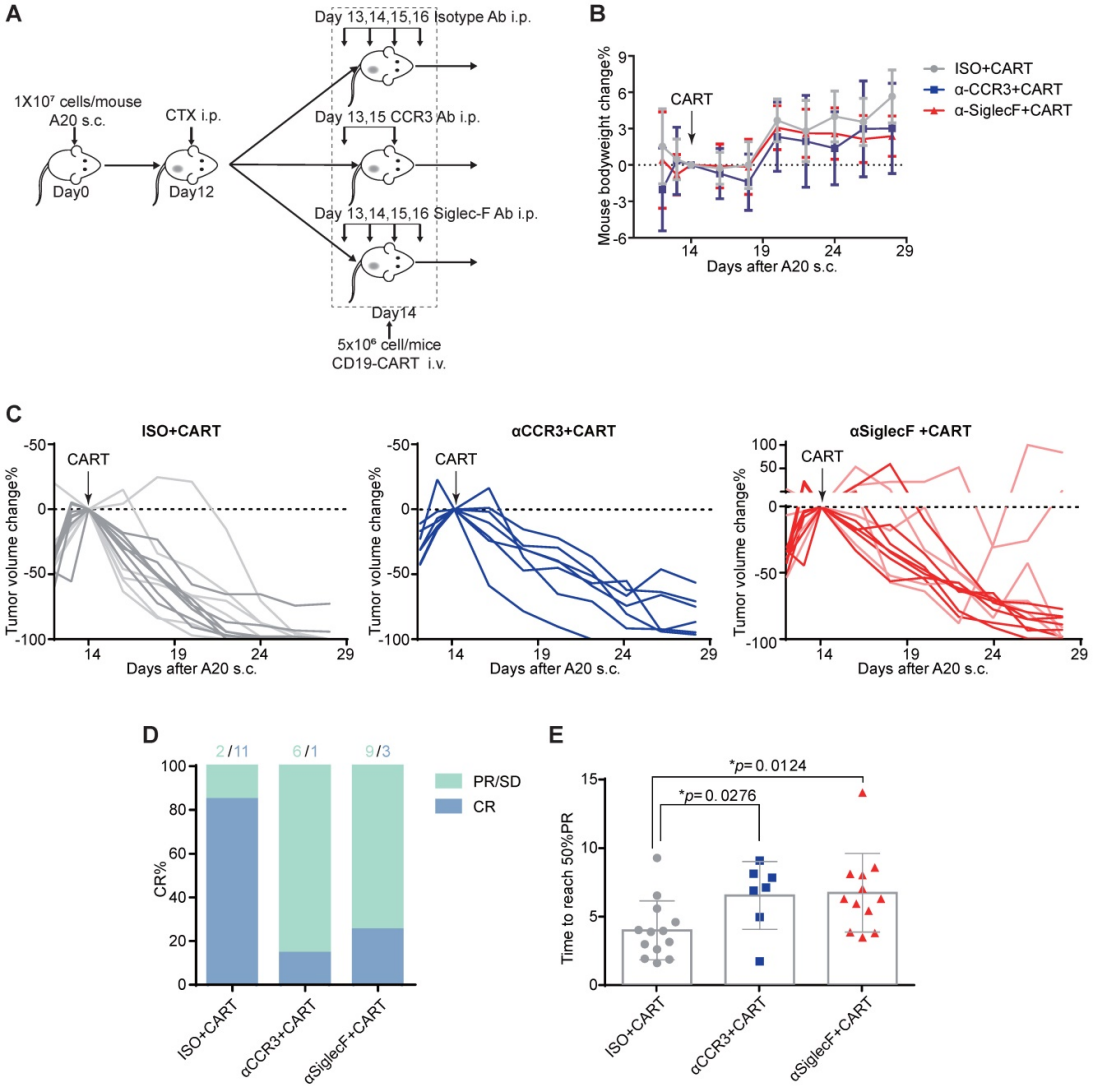
** Eosinophil depletion reduces anti-CD19 CAR-T cell antitumor efficacy. (A)** Schematic of the experiment. Ten million mouse lymphoma A20 (CD19^+^) cells were subcutaneously injected into each syngeneic BALB/c mouse on day 0. Each mouse was intraperitoneally administered 2 mg cyclophosphamide (CTX) on day 12. Each mouse was intraperitoneally administered 15 µg/day anti-mouse Siglec-F antibody or isotype control for 4 days starting on day 13. Each mouse was intraperitoneally administered 100 µg anti-mouse CCR3 antibody or isotype control on days 13 and 15. Each mouse was intravenously injected with 5 × 10^6^ anti-CD19 CAR-T cells on day 14. Body weights and tumor volumes were measured every 2 days. **(B)** Body weight changes (%) were monitored from day 12, as indicated. Data are shown as the mean ± SD. **(C)** A20 lymphoma growth in BALB/c mice treated with an isotype control antibody (left; n = 13; two experiments combined; light gray: 1^st^ round experiment; dark gray: 2^nd^ round experiment) or a CCR3 antibody (middle; n = 7) or Siglec-F antibody (right; n = 12 mice; two experiments combined; light red: 1^st^ round experiment; dark red: 2^nd^ round experiment). Arrows indicate CAR-T cell infusion.** (D)** Complete response (CR) rate in various groups. PR, partial response; SD, stable disease; PD, progressive disease. **(E)** Time to reach 50% partial tumor response in various groups. Isotype, gray, n = 13; anti-CCR3, blue, n = 7; anti-Siglec-F, red, n = 12. Data were analyzed by unpaired two-tailed *t*-tests and are shown as the mean ± standard deviation (SD). Tumor size at the time of CAR-T cell injection was considered the baseline.

The aforementioned clinical data indicate that patients with elevated peripheral eosinophil counts achieve better clinical outcomes in response to anti-CD19 CAR-T cell immunotherapy. We examined the mechanism driving the correlation between eosinophil counts and CAR-T cell efficacy by administering anti-CD19 CAR-T cell therapy to an immunocompetent mouse lymphoma model 10. We subcutaneously injected 10^7^ A20 B lymphoma cells into the flanks of BALB/c mice and allowed the tumors to reach an average volume of 400 mm^3^. The mice were preconditioned with cyclophosphamide (CTX; 2 mg/mouse) for acute lymphodepletion, similar to a clinical B-NHL protocol 11, 12. Anti-CD19 CAR-T cells, which are the most representative construct in the field of CAR-T cell therapies, were engineered to coexpress CAR and GFP separated by a P2A linker (**Figure S2**) and infused at 5×10^6^ cells/mouse 2 days after preconditioning (**Figure 3A**). To investigate the functional role of eosinophils in mediating tumor regression, we intraperitoneally injected an isotype control or anti-mouse CD193 (CCR3) antibody (clone# 6S2-19-4) 13 or anti-mouse CD170 (Siglec-F) antibody (clone #238047) 14 to deplete eosinophils* in vivo*. We monitored tumor size as a surrogate for treatment efficacy and body weight loss as an indicator of adverse toxicity.

CTX preconditioning induced immediate but temporary body weight loss caused by adverse gastrointestinal events. However, this side effect gradually abated in both treatment groups (**Figure 3B**). Anti-CD19 CAR-T cell administration eradicated A20 tumors in 13 eosinophil-competent, tumor-bearing mice. By day 28, 11 mice reached complete remission. In the eosinophil-depleted group, one out of seven mice treated with anti-CCR3 antibody were tumor-free by day 28. Three out of 12 mice treated with an anti-Siglec-F antibody were tumor-free, and two mice exhibited severe relapse and rapid tumor growth (**Figures 3C and 3D**). We compared the time to reach 50% partial tumor remission after CAR-T cell treatment. The interval was significantly shorter for eosinophil-competent mice than for antibody-depleted mice (**Figure 3E**). Comparable tumor growth was noted for mice subjected to eosinophil depletion but not infused with CAR-T cells (**Figure S3**). Therefore, eosinophils are functionally necessary for maximal anti-CD19 CAR-T cell therapeutic efficacy.

### Eosinophil depletion impairs intratumoral CAR-T cell recruitment

**Figure 4:**
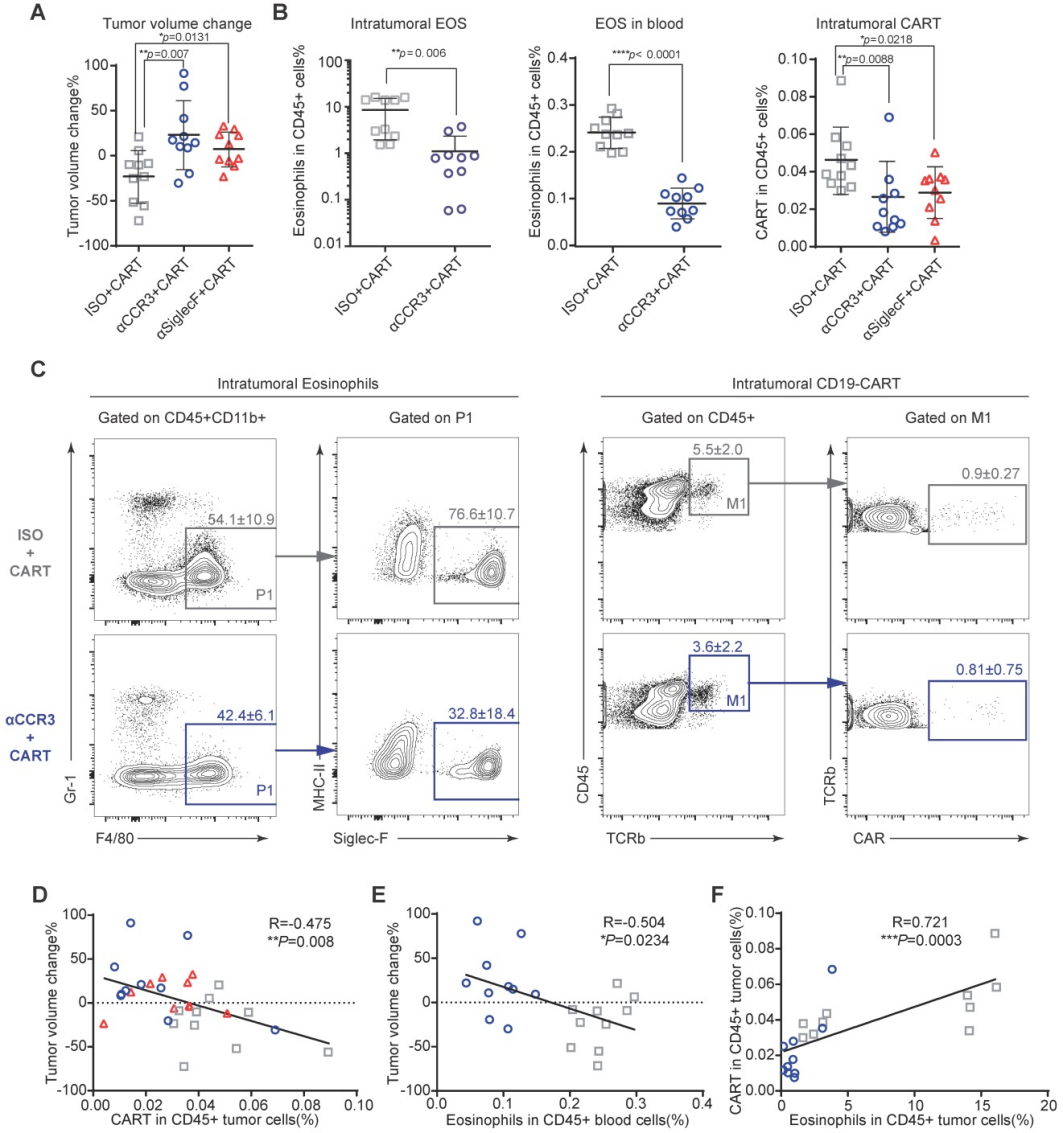
** Eosinophil depletion impairs intratumoral anti-CD19 CAR-T cell recruitment.** After A20 lymphoma establishment, BALB/c mice were intraperitoneally administered an anti-mouse CCR3 antibody, anti-mouse Siglec-F antibody or isotype control followed by anti-CD19 CAR-T cells (Figure S3A). **(A)** Percent A20 tumor volume change in BALB/c mice 3 days after anti-CD19 CAR-T cell infusion. Isotype, gray, n = 10; anti-CCR3, blue, n = 10; anti-Siglec-F, red, n = 10. Data are shown as the mean ± SD. **(B)** Statistical analyses of % intratumoral eosinophils, blood eosinophils, and tumor-infiltrating anti-CD19 CAR-T cells. Isotype, gray, n = 10; anti-CCR3, blue, n = 10; anti-Siglec-F, red, n = 10. Data are shown the mean ± SD. **(C)** Representative flow cytometry plots of intratumoral eosinophils and tumor-infiltrating anti-CD19 CAR-T cells. **(D)** Scatterplots depicting correlations between intratumoral anti-CD19 CAR-T cells and % tumor volume change after CAR-T cell infusion (n = 30). **(E)** Scatterplots depicting correlations between % tumor volume change and circulating eosinophils (n = 20). **(F)** Scatterplots depicting correlations between intratumoral eosinophils and anti-CD19 CAR-T cells (n = 20). Data in the left panels **(B)** were analyzed by unpaired two-tailed Welch's *t*-test. Data in **(D-F)** were analyzed by Pearson's correlation.

To investigate the mechanism by which eosinophils improve anti-CD19 CAR-T cell therapeutic efficacy, we performed immune profiling of A20 mouse lymphomas 3 days after CAR-T cell infusion (**Figures S4A and S5**). CAR-T cell treatment efficacy was evident in mice with intact eosinophils. Tumors in mice treated with an isotype control antibody were significantly smaller than those in eosinophil-depleted mice administered an anti-CCR3 antibody (**Figure 4A;** **P = 0.007) and anti-Siglec-F antibody (**Figure 4A**; *P = 0.0131). The anti-Siglec-F depletion antibody (R&D, Clone # 238047) and flow antibody (BD, Clone # E50-2440) competitively bind antigen. Thus, flow cytometry analysis did not accurately reflect the number of eosinophils remaining after anti-Siglec-F antibody treatment. Hence, eosinophil-related data for the Siglec-F antibody-treated group were not included in this analysis. Flow cytometry analysis of tumor, blood, spleen, and bone marrow (BM) samples demonstrated that anti-CCR3 antibody administration depleted most circulating CD45^+^CD11b^+^Gr-1^lo^F4/80^+^Siglec-F^+^MHC-II^-^ eosinophils (**Figure 4B left and middle** and **Figure S4B**). Substantially fewer CAR-T cells were recovered from the tumors in eosinophil-depleted mice (**Figures 4B right and 4C**; **P = 0.0088 for isotype vs. anti-CCR3; *P = 0.0218 for isotype vs. anti-Siglec-F; **Figure S6**). Changes in tumor volume were negatively correlated with CAR-T cell intratumoral infiltration (**Figure 4D**; **P = 0.008, R = -0.475) and eosinophil counts in peripheral blood (**Figure 4E**; *P = 0.0234, R = -0.504). We intravenously transferred 10^6^ activated eosinophils into tumor-bearing mice 1 day before CAR-T cell treatment followed by the intravenous transfer of 5×10^6^ CAR-T cells. CAR-T cells combined with eosinophil transfer showed a tendency toward more effective tumor eradication than CAR-T cells alone (**Figure S7**). The link between eosinophil abundance and CAR-T cell efficacy observed here was identical to the relationship discovered in human B-NHL trials (**Figure 1**). Therefore, our preclinical mouse model closely resembled human clinical outcomes. Moreover, mouse intratumoral eosinophil counts were positively associated with CAR-T cell infiltration (**Figure 4F;** ***P = 0.0003, R = 0.721). To elucidate the molecular mechanism underlying the promotion of intratumoral infiltration by eosinophils, we examined the expression of activated T cell attractants such as CXCL9 and CXCL10 in tumors with competent eosinophils and those with antibody-mediated depletion (**Figure S8**). We identified the significant downregulation of these attractants (**P = 0.0092 for CXCL9 and *P = 0.0235 for CXCL10) in eosinophil-depleted tumors. This finding was consistent with established eosinophil characteristics 14.

The above data suggest that like adoptively transferred effector T cells 7, eosinophils infiltrate tumors and actively recruit CAR-T cells. This mode of action might explain the capacity of peripheral eosinophils to serve as baseline biomarkers predicting the clinical responsiveness of B-NHL to anti-CD19 CAR-T cell therapy.

## Discussion

Eosinophils have been recognized as innate immune cells with nonspecific cytolytic activity against tumor cells 15. However, recent evidence from animal models has revealed a more comprehensive antitumor mechanism of eosinophils. At tumor sites, eosinophils repolarize macrophages, normalize tumoral blood vessels, and recruit and activate tumor-specific cytotoxic T cells via a CXCL9/CXCL10-dependent mechanism 7. Here, we showed that at baseline, the absolute number of eosinophils in peripheral blood is positively correlated with patient clinical outcomes. Our regular follow-up surveillance revealed that patients with high baseline eosinophil counts had significantly higher objective responses and that high baseline eosinophil counts were associated with durable PFS. The precise high eosinophil threshold remains to be defined in larger cohorts. Nevertheless, we propose that eosinophil counts are potential biomarkers for ≥ 3-mo PFS in B-NHL patients.

Previous biomarkers were based on complex immunophenotyping or high-throughput analysis 16. In contrast, preinfusion baseline eosinophil measurements are inexpensive and widely accessible. Both of our B-NHL cohorts comprised small numbers of patients. However, the predictive efficacy of eosinophils in anti-CD19 CAR-T cell therapy was comparable to those of PD-L1 expression (AUC = 56%; 95% CI = 48-82%) and total mutational burden (AUC = 66%; 95% CI = 45-89%) used for anti-PD1 blockade therapies in melanoma patients 17. In an A20 tumor-bearing mouse model, we demonstrated a clinically meaningful association between eosinophil infiltration and the efficacy of CAR-T cell immunotherapies. An additional B-cell lymphoma mouse model is needed to further strengthen this association. In regard to the clinical evidence, despite further support from two independent validation cohorts, we understand that our limited cohort size cannot fully demonstrate the predictive power of eosinophils or determine the predictive power for specific pathologic types in our cohorts. Additionally, due to the unavailability of immunophenotyping data of eosinophils in clinical practice, we could not identify whether only activated eosinophils are relevant for predicting the efficacy of adoptive transfer therapies.

The correlation between the eosinophil count and CAR-T cell therapeutic efficacy could help inform clinical decisions by helping to predict patient responses and guiding the administration of alternative therapies. Our retrospective analysis provided evidence that baseline eosinophil counts correlate with distinct clinical outcomes for B-NHL patients who are administered anti-CD19 CAR-T cell immunotherapy. The application of this standard blood assay to identify responders may improve patient selection criteria. Based on the cytolytic activity and potential of eosinophils to attract T cells 18, this clinical evidence may also stimulate the development of new strategies to improve the efficacy of adoptive cell transfer immunotherapies by bolstering eosinophils in further studies.

## Methods

### Study design and participants

For the discovery cohort (Cohort 1), patients were enrolled in a clinical trial to evaluate the safety and efficacy of anti-CD19 CAR-T cells in relapsed or chemotherapy-refractory B-cell lineage non-Hodgkin malignancies. The present study was a retrospective analysis of patients in the IL-2 arm of the aforementioned clinical trial. The study was registered at ClinicalTrials.gov (NCT02652910). Patients were enrolled from November 2015 to June 2017 at the Department of Oncology of the Second Affiliated Hospital (Xinqiao Hospital) at Army Medical University (Cohort 1; Discovery Cohort). Two independent validation cohorts were also included in this study. Cohort 2 (Validation Cohort 1) 19 was used to investigate CD19-BBz(86) CAR-T cell therapy in patients with B-cell lymphoma. Patients were enrolled from June 2016 to August 2017 at Beijing Cancer Hospital. Four patients were excluded because of inadequate blood assay timepoints. The ClinicalTrials.gov identifier was NCT02842138. Cohort 3 (Validation Cohort 2) 20 was used to examine tandem CD19/CD20 CAR-T cell therapy in refractory/relapsed B-cell lymphoma. Patients were enrolled from May 2017 to September 2018 at the Department of Biotherapeutics of The First Medical Center at the Chinese PLA General Hospital. The ClinicalTrials.gov identifier was NCT03097770. All enrolled patients provided written informed consent, and baseline blood samples were obtained immediately before preconditioning chemotherapy. No patients presented with any infectious or autoimmune disease or chemotherapy-induced myelosuppression at the time of baseline sampling. Clinical responses to CAR-T cell therapy were assessed according to the Lugano criteria 21. The protocol was approved by the IRB of the Second Affiliated Hospital at Army Medical University, Chongqing, China, the Medical Ethics Committee of Beijing Cancer Hospital, and the Ethics Committee of the Chinese PLA General Hospital.

### Manufacturing of human CAR-T cells

Patients were enrolled in this study after eligibility screening and confirmation. They underwent leukapheresis to obtain peripheral blood mononuclear cells (PBMCs), which were then frozen and shipped to the central cell processing facility. For Cohort 1, > 10^7^ CD3^+^ T cells obtained by anti-CD3/28 Dynabead separation were stimulated with IL-2 for 48 h and transduced with a retroviral vector containing anti-CD19-CD28ζ CAR. Anti-CD19 was a single-chain variable fragment (scFv) derived from an FMC63 monoclonal antibody. The CAR-T cells were expanded, and the partially completed product was washed and cryopreserved to formulate the final drug product. After identity, % CAR expression, T cell phenotype, potency, and sterility quality control (QC) testing, the final product was shipped to the clinical center either fresh or at ≤ -150 °C. For Cohort 2, the anti-CD19 CAR consisted of an FMC63 scFv clone, CD8α extracellular and transmembrane domains, and 4-1BB and CD3ζ cytoplasmic domains. For Cohort 3, a tandem CAR construct was generated by combining the CD19 scFv derived from an FMC63 monoclonal antibody and the CD20 scFv derived from a Leu-16 monoclonal antibody. A hinge, CD8α transmembrane domains, and 4-1BB and CD3ζ cytoplasmic domains were used in the tandem construct. CAR-T cell preparation and quality control details for Cohorts 2 and 3 were previously described 19, 20.

### Human lymphodepletion chemotherapy

In Cohort 1, endogenous lymphocytes were depleted before adoptive CAR-T cell transfer with chemotherapeutic preconditioning regimens consisting of 800-1,000 mg/m^2^ intravenous CTX on day 1 and 35 mg/m^2^ intravenous fludarabine on days 1-3. In Cohort 2, preconditioning chemotherapy included 3 days of fludarabine (25 mg/m^2^ on days 1-3) and CTX (250 mg/m^2^ on days 1-3). In Cohort 3, preconditioning chemotherapy comprised CTX (20-30 mg/kg over 3 days) and fludarabine (20-30 mg/m^2^ on days 1-3) with or without doxorubicin liposomes (10 mg/m^2^ on day 1).

### Human CAR-T cell infusion

Anti-CD19 CAR-T cells were intravenously administered to each patient 2 days after the completion of preconditioning chemotherapy. CAR-T cells were synthesized for all enrolled patients. In Cohort 1, 12 patients were administered cryopreserved CAR-T cells, whereas the other four patients were administered fresh CAR-T cells. In Cohorts 2 and 3, all 49 patients were administered fresh CAR-T cells.

### Cell culture

Mouse A20 lymphoma cells were cultured in RPMI 1640 medium supplemented with 10% (v/v) fetal bovine serum (FBS; Thermo Fisher Scientific, Waltham, MA, USA), 1% (w/v) penicillin/streptomycin (Sigma-Aldrich Corp., St. Louis, MO, USA), 2 mM *L*-glutamine (Thermo Fisher Scientific, Waltham, MA, USA), 1 mM sodium pyruvate (Thermo Fisher Scientific, Waltham, MA, USA), and 0.05 mM 2-mercaptoethanol (Sigma-Aldrich Corp., St. Louis, MO, USA). HEK293T and 3T3 cells were cultured in Dulbecco's modified Eagle's medium (DMEM) supplemented with 10% (v/v) FBS (Thermo Fisher Scientific, Waltham, MA, USA) and 1% (w/v) penicillin/streptomycin (Sigma-Aldrich Corp., St. Louis, MO, USA). All cells were purchased from Duke University Cell Culture Faculties (Durham, NC, USA), confirmed to be mycoplasma-free, and cultured in a humidified 37 °C incubator with 5% CO_2_.

### Retroviral mouse model construction

The MP71 retroviral plasmid served as the backbone 22. Anti-CD19 CAR-T cells were constructed as previously described 23. Anti-mouse CD19 scFv was derived from the mouse CD19 antibody ID3 followed by the partial CD28 and the CD3ζ signaling domain. The first and third CD3ζ immunoreceptor tyrosine-based activation motifs (ITAMs) were inactivated. A P2A sequence was linked to GFP, and the latter served as a marker for mouse anti-CD19 CAR-T cells.

### Mouse CAR-T cell fabrication

MP71 and pclEco were cotransfected with Lipofectamine^TM^ 2000 (Thermo Fisher Scientific, Waltham, MA, USA) into 293T cells. Supernatants containing viral particles were collected 48 h after transfection and passed through a 0.45-µm filter. Mouse lymph nodes were excised, digested, and pooled in a single-cell suspension. Lymphocytes were stimulated with 4 µg/mL anti-CD3 and 4 µg/mL anti-CD28 antibodies precoated on cell culture plates. The lymphocytes were cultured in RPMI 1640 medium supplemented with 10% (v/v) FBS, 1% (w/v) penicillin/streptomycin, 2 mM *L*-glutamine, 1 mM sodium pyruvate, 0.05 mM 2-mercaptoethanol, 0.1 mM nonessential amino acids, and 50 U/mL human IL-2 (PeproTech Inc., Rocky Hill, NJ, USA) for 36 h. The cells were then spin-infected with virus at 2,500 rpm and 32 °C for 2 h in the presence of 6 µg/mL Polybrene (Sigma-Aldrich Corp., St. Louis, MO, USA). Subsequent experiments were conducted 48 h after infection.

### Preclinical mouse model

Each BALB/c mouse aged 6-10 wks was subcutaneously injected with 10^7^ A20 cells on day 0. After 12 days, each mouse was preconditioned with 2 mg CTX (Baxter Oncology GmbH, Nordrhein-Westfalen, Germany) followed by 15 µg/mouse/day anti-Siglec-F (R&D MAB17061) or isotype control (R&D MAB006) antibody administration for 4 days starting on day 13. Alternatively, each mouse was administered 100 µg anti-CCR3 (Bioxcell 6S2-19-4) antibody on days 13 and 15. Then, 5 × 10^6^ anti-CD19 CAR-T cells were infused into the tail vein of each mouse on day 14. Mouse body weight changes and tumor sizes were closely monitored every 2 days after treatment.

Mice used in fluorescence-activated cell sorting (FACS) analysis were treated as previously described except they were sacrificed on day 17 to obtain the organs and tissues required for the procedure. For the eosinophil transfer experiment, eosinophils were obtained and activated with TNF-α and IFN-γ as previously described 14. One million activated eosinophils were intravenously injected into each tumor-bearing mouse on day 13. These mice had undergone CTX preconditioning on day 12. Each mouse was intravenously injected with 5×10^6^ anti-CD19 CAR-T cells on day 14.

Tumor dimensions were measured with a caliper, and tumor volumes were calculated as ½(length × width^2^). Mice were purchased from The Jackson Laboratory (Bar Harbor, ME, USA) and maintained under specific pathogen-free conditions at the Animal Facility of Duke University (Durham, NC, USA). All mouse studies were performed in accordance with the guidelines and protocols approved by the Duke University Animal Care and Use Committee (Durham, NC, USA).

### FACS analysis

Human CAR-T cells in peripheral blood were assessed by flow cytometry and stained with fluorescently labeled antibodies against CD3, CD4, CD8, CD19, and CD10 (BioLegend, San Diego, CA, USA). CAR was detected with biotin-labeled polyclonal goat anti-mouse F(ab)_2_ antibodies (anti-Fab; Jackson ImmunoResearch, West Grove, PA, USA) and BV421-labeled streptavidin (BioLegend, San Diego, CA, USA). Flow cytometry was performed with a MACSQuant Analyzer 10 (Miltenyi Biotec GmbH, Bergisch Gladbach, Germany) and FlowJo v. 10 (Treestar, Inc. Ashland, OR, USA).

The mice were sacrificed, and their spleens, BM, blood, draining lymph nodes, and tumors were harvested and processed as single-cell suspensions. Red blood cells were lysed in 1× ammonium-chloride-potassium (ACK) buffer (Thermo Fisher Scientific, Waltham, MA, USA) for 1 min. Samples were stained with Aqua live/dead dye (Invitrogen, Carlsbad, CA, USA) according to the manufacturer's protocol. Fc receptors were blocked with an anti-mouse CD16/CD32 antibody (BD #553141) before surface staining with mTCRb PerCP-Cy5.5 (BioLegend #109227), mCD8b BV711 (BioLegend #126633), mCD45 APC (BioLegend #103112), mSiglec-F BV421 (BD #562681), mCD11b PECY7 (BioLegend #101216), Gr-1 AF700 (BioLegend #108422), mF4/80 PE (BioLegend #123110), and MHCII APCCY7 (BioLegend #107628). Data were acquired with an LSR Fortessa X20 (BD Biosciences, Franklin Lakes, NJ, USA) and analyzed with FlowJo v. 10 (Treestar, Inc. Ashland, OR, USA).

For intracellular cytokine staining, Golgi-blocking monensin and brefeldin A (Thermo Fisher Scientific, Waltham, MA, USA) were added 4 h before fixation in 2% (v/v) paraformaldehyde/phosphate-buffered saline (PFA-PBS). The cells were permeabilized in permeabilization buffer (Invitrogen, Carlsbad, CA, USA) according to the manufacturer's protocol. Samples were stained with anti-mouse CD8b PE (BioLegend #126608) and anti-mouse INF-γ Pacific Blue (BioLegend #505818). Flow cytometry was conducted with a BD FACSCanto^TM^ II analyzer (BD Biosciences, Franklin Lakes, NJ, USA).

### Statistical analysis

Unless otherwise specified, an unpaired twotailed *t*-test was used to compare the means of two experimental groups. An unpaired twotailed Welch's *t*-test was used when the sample variances were unequal. Correlation was analyzed by Pearson's correlation test. Survival curves were plotted with the Kaplan-Meier method. Statistical comparisons were made with the log-rank (Mantel-Cox) test. The ROC curve was plotted in the “pROC” package of R (R Core Team, Vienna, Austria). *P* < 0.05 was considered statistically significant.

## Supplementary Material

Supplementary figures.

Supplementary table 1.

Supplementary table 2.

Supplementary table 3.

## Abbreviations

ALL: acute lymphoblastic leukemia
AUC: area under the curve
B-NHL: B-lineage non-Hodgkin lymphoma
BM: bone marrow
CAR-T cells: chimeric antigen receptor T cells
CR: complete response
CRS: cytokine release syndrome
CTX: cyclophosphamide
DLBCL: diffuse large B-cell lymphoma
EOS: eosinophil
FL: follicular lymphoma
HR: hazard ratio
MZL: marginal zone lymphoma
PD: progressive disease
PFS: progression-free survival
PR: partial response
ROC: receiver operating characteristic
SD: stable disease
SPD: sum of the product of the perpendicular diameters
