# Supplementary material for: Peripheral eosinophil counts predict efficacy of anti-CD19 CAR-T cell therapy against B-lineage non-Hodgkin lymphoma
Source: Theranostics. 2021 Mar 4;11(10):4699–709. doi: 10.7150/thno.54546 (PMC7978305; doi:10.7150/thno.54546)
Supplement: Supplementary file 1 — Supplementary figures. [file thnov11p4699s1.pdf]

Supplementary Materials

Figure S1. Diagram for screening strategies.

Figure S2. Anti-CD19 CAR-T cells specifically react with $CD19^+$ tumor cells *in vitro*.

Figure S3. Tumor growth for mice receiving antibody-mediated eosinophil depletion and without CAR-T infusion

Figure S4. Anti-CCR3 antibody and anti-Siglec-F antibody deplete eosinophils *in vivo* separately.

Figure S5. Flow cytometry gating strategy for eosinophils and anti-CD19 CAR-T cells.

Figure S6. Fewer CAR-T cells were recovered from the tumors treated with eosinophils depletion antibody.

Figure S7. Tumor volume change in CD19-CART treated mice with/without eosinophil transferring.

Figure S8. Expression of intratumoral T-cell attractants

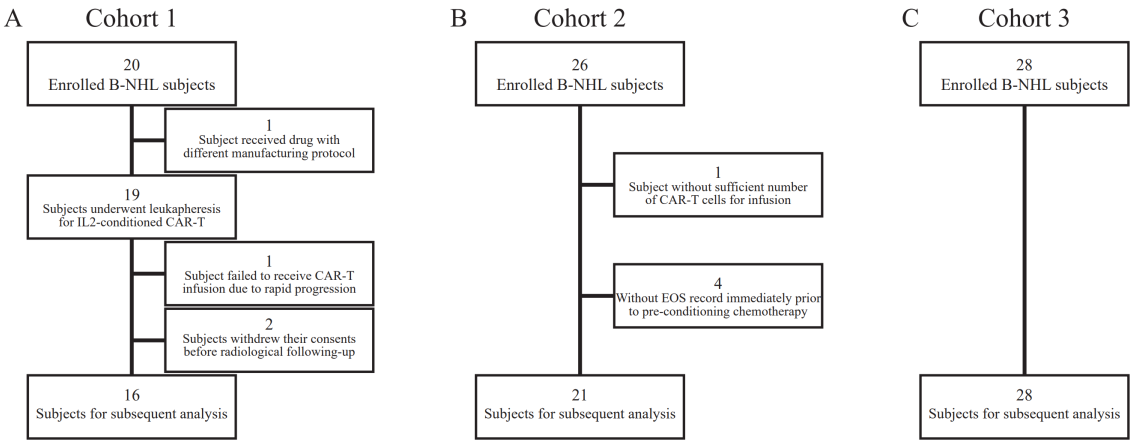

Figure S1. Diagram for screening strategies. Diagrams showed the screening strategies for identifying candidates for evaluating the predictive significance of baseline eosinophils. **(A)** Cohort 1; **(B)** Cohort 2; **(C)** Cohort 3.

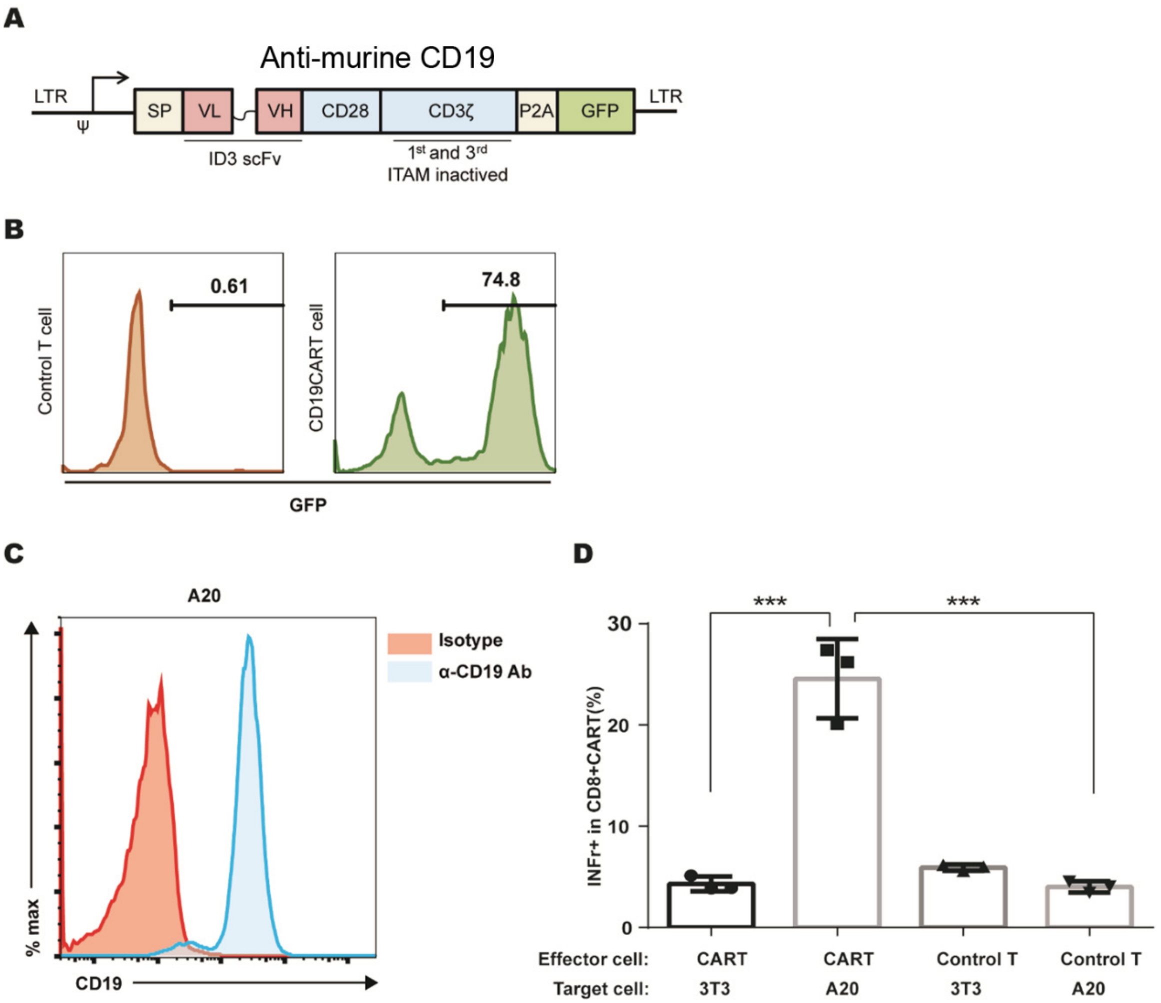

Figure S2. Anti-murine CD19 CAR-T cells specifically react with CD19^{+} tumor cells *in vitro*. (A) Diagram of the DNA encoding the 1D3-28Z CAR (ψ, retroviral packaging signal). **(B)** Prior to the mouse infusion procedure outlined in Fig. 3A, anti-murine CD19 CAR-T cells were tested for CAR expression. **(C)** Expression of CD19 on A20 lymphoma cells. **(D)** Specific reactivity of anti-CD19 CAR-T cells against CD19^{+} A20 lymphoma cells. Anti-CD19 CAR-T cells (4×10^{5}/ml) were co-cultured with A20 or 3T3 cells (CD19^{-}) at a ratio of 1:1 for 12 hours, then intracellular IFN-γ staining assays were conducted. IFN-γ expression in CAR-T cells co-cultured with A20 cells was significantly higher compared with 3T3 cell co-culture, demonstrating CAR-T specificity against CD19. n = 3 replicates for each group; *$P < 0.05$; ***$P < 0.001$ by unpaired *t*-test)

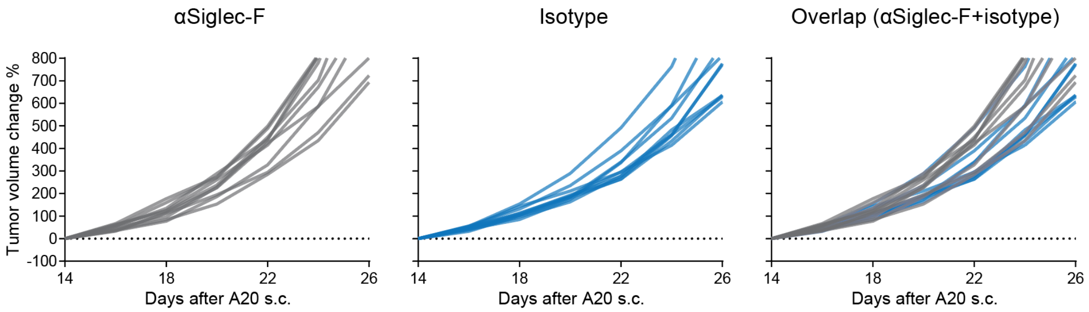

Figure S3. Tumor growth for mice receiving antibody-mediated eosinophil depletion and without CAR-T infusion. Mice were prepared as illustrated in Figure 3A. Infusion of CD19-CART i.v. on day 14 was replaced by saline. Each line represents one mouse in experiment.

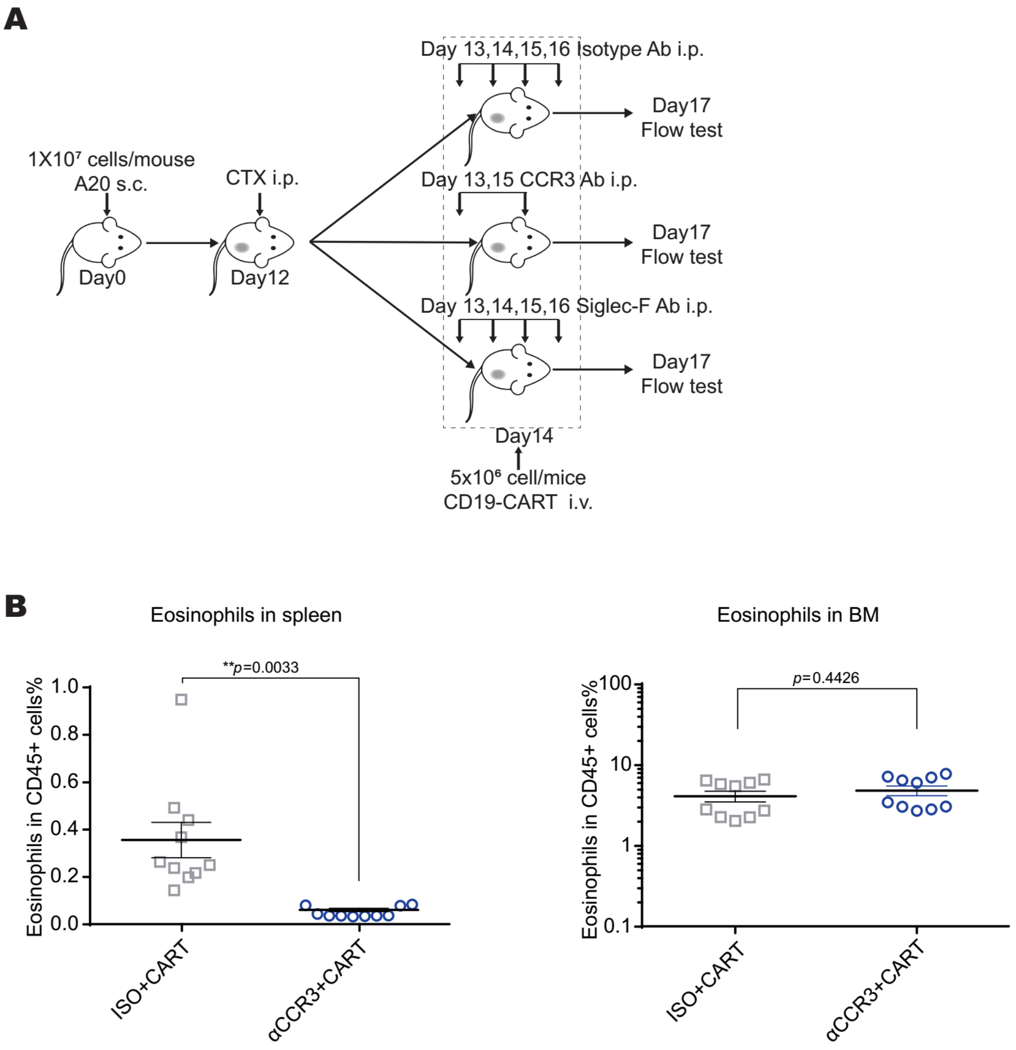

Figure S4. Anti-CCR3 antibody and anti-Siglec-F antibody deplete eosinophils in vivo separately. (A) Experimental schema for analyzing eosinophil and CAR-T cell counts after eosinophil depletion. **(B)** Eosinophil percentages among $CD45^+$ splenocytes (left) and bone marrow (right) for isotype antibody, CCR3 antibody and Siglec-F antibody treated groups. Isotype, gray, n = 10; anti-CCR3, blue, n = 10; Data in left panels in (B) were analyzed by unpaired two-tailed Welch's t-test.

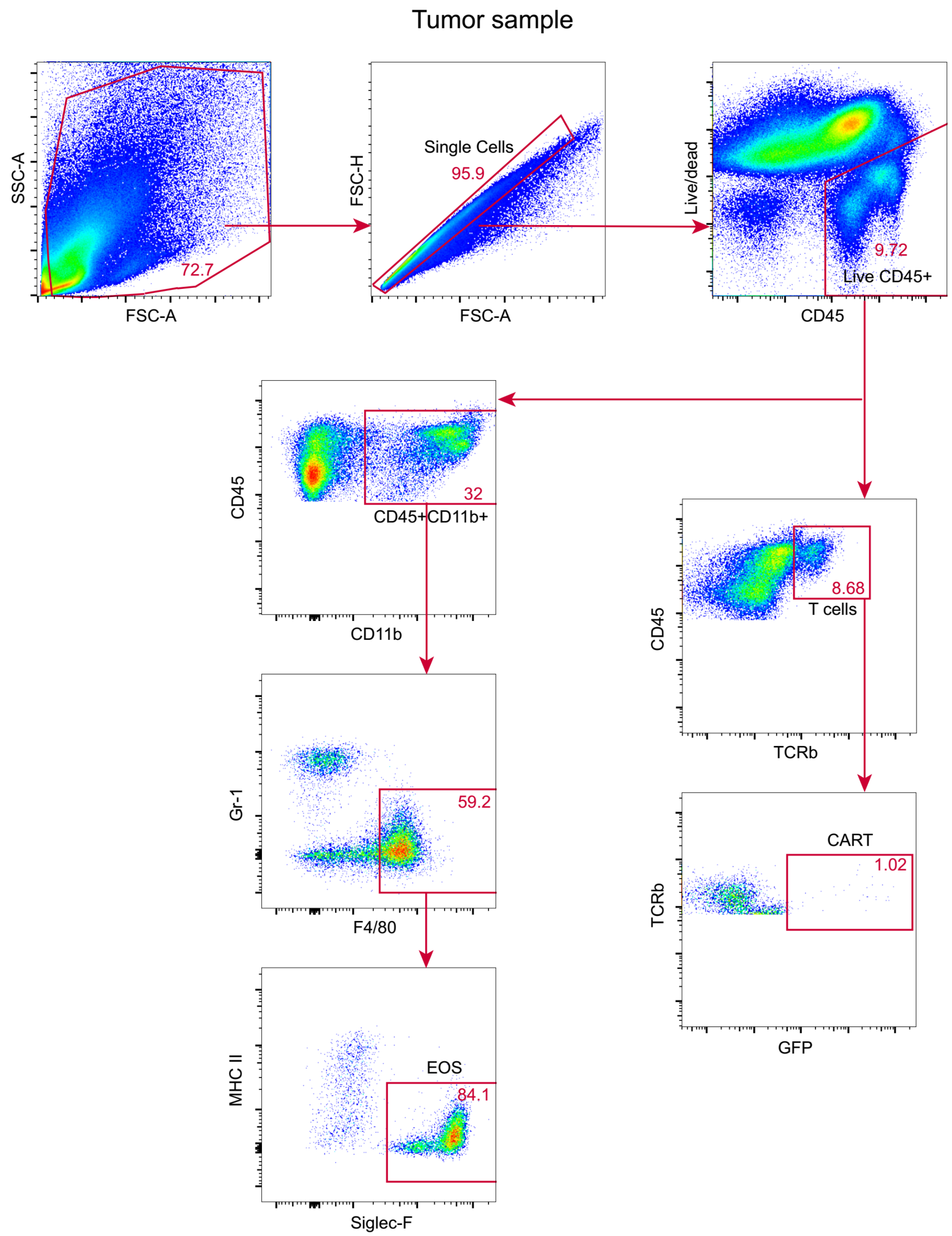

Figure S5. Flow cytometry gating strategy for eosinophils and CD19 CART cells. Tumor samples were gated on FSC-A versus SSC-A followed by a singlet gate excluded any doublets, then a viability CD45^{+} gate was applied to exclude any dead cells. CD19-CART cells were assessed by TCRb and GFP double positive group. Eosinophils were first gated from CD45^{+} live cells, and then CD11b^{+}Gr-1^{lo}F4/80^{+}Siglec-F^{+}MHCII^{-} groups were analyzed as eosinophils.

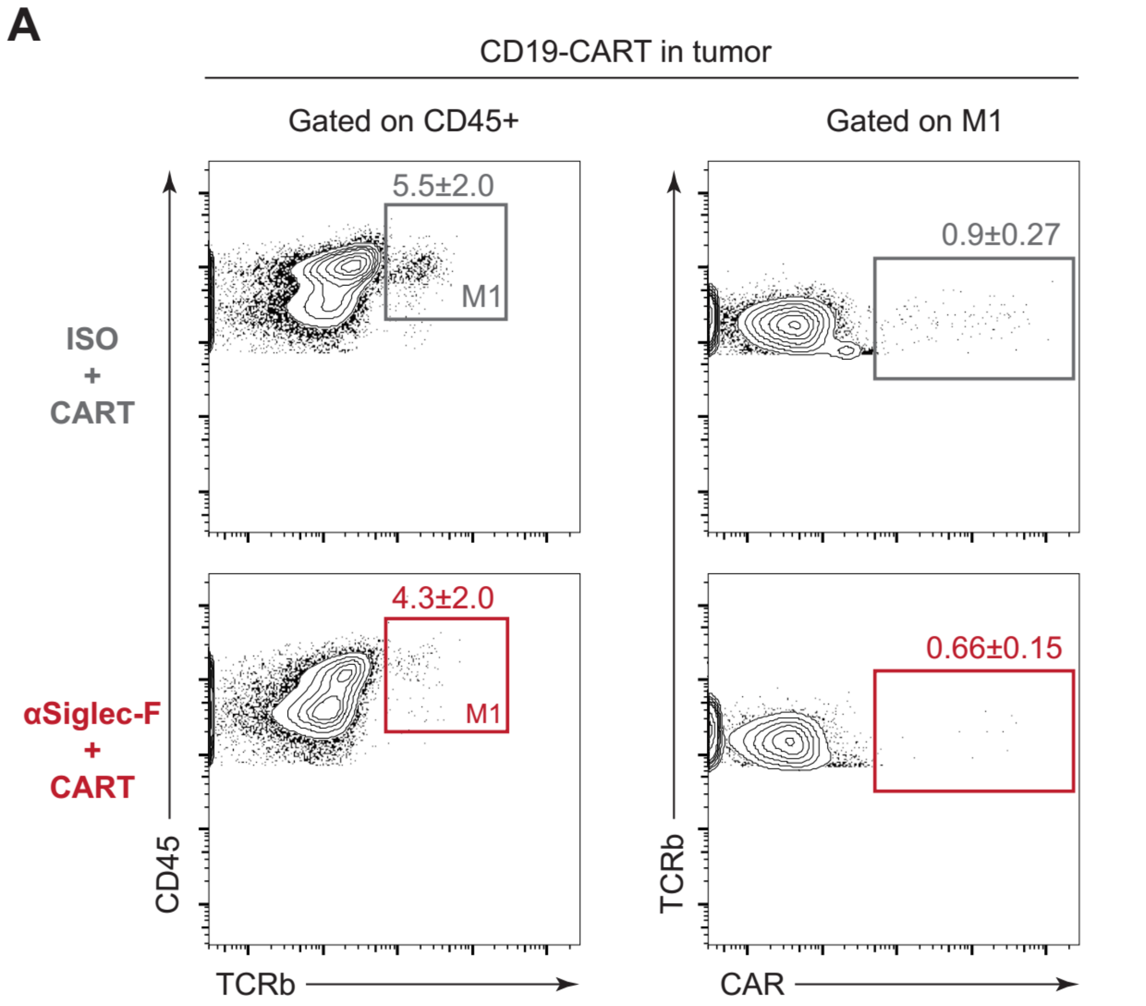

Figure S6. In these eosinophil-depleted animals, markedly fewer CAR-T cells were recovered from the tumor. Representative flow cytometry plots of tumor-infiltrating T cells (left) and intratumoral anti-CD19 CAR-T cells (right). Data are shown as means ±SD.

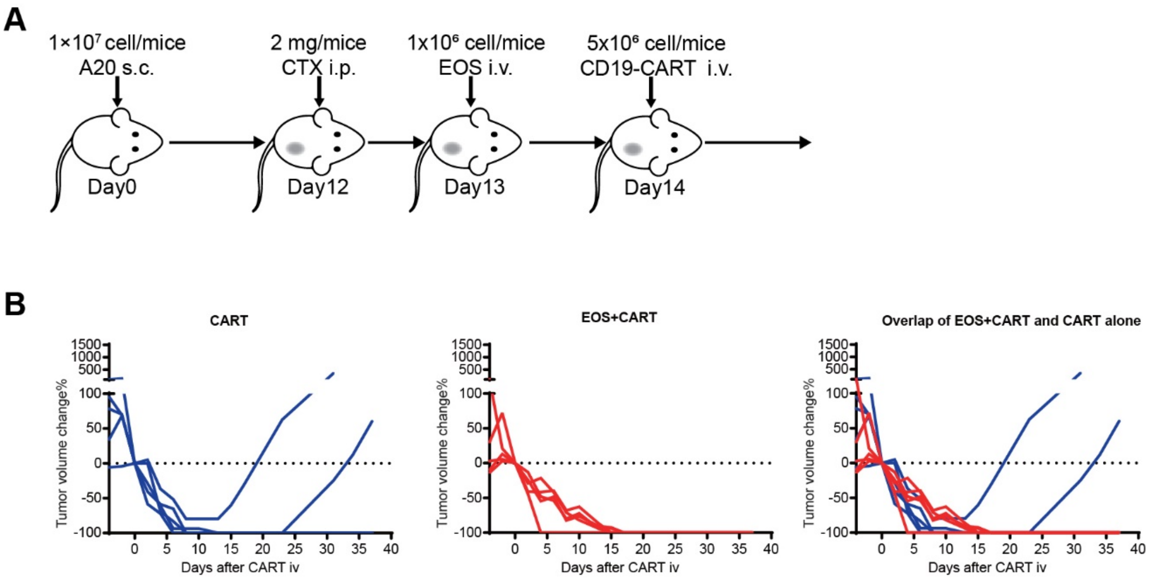

Figure S7. Tumor volume change percentage in CD19-CART treated mice with/without eosinophil transferring. (A) Experimental schema for eosinophils transferring and CAR-T cells treatment in tumor-bearing mice. Balb/c mice were subcutaneously injected with 1×10^7 A20 lymphoma cells. 12 days later, all the mice were treated with 2mg/mouse CTX for preconditioning, following with/without 1×10^6 activated eosinophils intravenously injection on day13. 5×10^6 CD19-CART cells were injected into mice on day14 (Arrow). **(B)** Tumor volume change percentage in mice transferred with eosinophils or without eosinophils. Blue, mice were transferred with CD19-CART only; red, mice were transferred with eosinophils and CD19-CART cells. (n=5 per group, tumor size at the time point of CART injection was regard as baseline.)

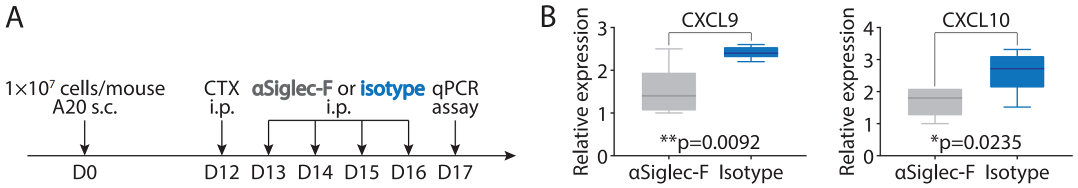

Figure S8. Expression of intratumoral T-cell attractants. (A) Experimental schema. Ten million mouse lymphoma A20 ($CD19^{+}$) cells were subcutaneously injected into syngeneic *BALB/c* mice on day 0. Each mouse was intraperitoneally administered 2 mg cyclophosphamide (CTX) for preconditioning on day 12. Each mouse was intraperitoneally administered 15 µg/d anti-mouse Siglec-F antibody or isotype control for 4 d starting on day 13. Intratumoral CXCL9 and CXCL10 expression levels were measured by qPCR on day 17. **(B)** Boxplots showing relative CXCL9 and CXCL10 expression in Siglec-F- or isotype-treated tumors (n = 5). Horizontal line: median expression level. Statistical analysis consisted of Mann-Whitney *U*-test. qPCR primers were CXCL9 forward: CTTTTCCTCTTGGGCATCAT; CXCL9 reverse: GCATCGTGCATTCCTTATCA; CXCL10 forward: GACGGTCCGCTGCAACTG; and CXCL10 reverse, CCCTATGGCCCTCATTCTCA.
